# Pharmacokinetic Changes and Dosing Modification of Aminoglycosides in Critically Ill Obese Patients: A Literature Review

**DOI:** 10.14740/jocmr1858w

**Published:** 2014-05-22

**Authors:** Dimitrios Velissaris, Vasilios Karamouzos, Markos Marangos, Charalampos Pierrakos, Menelaos Karanikolas

**Affiliations:** a Internal Medicine Department, University Hospital of Patras, Rion 26500, Greece; b Intensive Care Department, Brugmann University Hospital, Brussels 1030, Belgium; c Department of Anesthesiology, Washington University School of Medicine, St. Louis, Missouri, USA

**Keywords:** Aminoglycoside, Gentamicin, Amikacin, Tobramycin, Obesity, Sepsis, Critical illness, Pharmacokinetics

## Abstract

The objective of the paper is to review the literature and provide recommendations for use of aminoglycoside antibiotics in critically ill obese patients. Literature search in PubMed for all articles on the use of aminoglycosides in critically ill obese patients was conducted, and all articles related to pharmacokinetics in obesity were reviewed. Bibliographies of all searched manuscripts were also reviewed in an attempt to find additional references. Although aminoglycoside pharmacokinetics have been described in detail, data on aminoglycoside use and appropriate dose modification in critically ill obese patients are very limited. Knowledge on aminoglycoside pharmacokinetics and use in critically ill obese patients is incomplete. Pathophysiologic changes in obesity can result in sub- or supra-therapeutic aminoglycoside plasma concentrations, especially in the presence of sepsis. Rigorous clinical studies are needed to establish aminoglycoside dosing guidelines in critically ill obese patients with sepsis.

## Introduction

Obesity is a major worldwide health problem, and is associated with serious diseases and increased morbidity and mortality. Physiologic changes in obesity significantly influence antibiotic pharmacokinetic characteristics, including distribution, protein binding, metabolism and renal excretion. In order to reach therapeutic levels, some medications require dosing adjustments in obese patients, especially in the presence of critical illness. Aminoglycosides act synergistically with other antimicrobial agents, and are very important for treatment of serious infections. However, because aminoglycoside plasma levels can be significantly affected by obesity, due to aminoglycoside distribution into adipose tissue, dosing modification is needed in order to reach therapeutic plasma levels, and therapeutic drug monitoring (TDM) has been recommended. The aim of this study is to review data on aminoglycoside pharmacokinetics in obesity and summarize published data regarding the use of aminoglycosides and the need for dose modification in critically ill obese patients with sepsis.

## Literature Search Methods

We searched for publications relevant to this review, using the PubMed database. The initial PubMed search was conducted in September 2012 and was updated in October 2013. The search was conducted using the terms “aminoglycosides dosing and obesity”, “aminoglycosides pharmacokinetics and obesity”, “aminoglycosides in sepsis and obesity” and “aminoglycosides pharmacokinetics in sepsis” as keywords. We excluded animal studies, and studies published in languages other than English. There was no exclusion based on type of aminoglycoside or publication date. The search included all types of articles, including case reports and review articles, and the bibliography of all extracted manuscripts was reviewed in attempt to identify additional references. All articles published in English related to aminoglycoside use in obesity were reviewed. When we identified publications of overlapping data, we only used data from the newer or more detailed publication. Two authors reviewed the abstract and text of all articles that seemed relevant to this review.

## Literature Review

Our literature search revealed 20 articles in the “aminoglycosides dosing and obesity” term, 42 articles in the “aminoglycosides pharmacokinetics and obesity”, 165 articles in the “aminoglycosides pharmacokinetics in sepsis” and seven articles in the “aminoglycosides in sepsis and obesity”. We then searched for publications with text combining most keywords. The combination of the above searches revealed a total of 33 articles, case series and letters which were included in this review.

Overall, we found very limited data on aminoglycoside pharmacokinetics and dose modification in obesity. Four early studies on morbidly obese patients, by Schwartz referring to gentamicin and tobramycin in 1978 [1], by Blouin referring to tobramycin in 1979 [2], by Bauer referring to amikacin in 1980 [3] and by Korsager referring to gentamicin in 1980 [4] concluded that morbidly obese patients have larger volume of distribution (Vd) compared to normal weight patients.

Schwartz et al evaluated the pharmacokinetics of gentamicin and tobramycin and calculated elimination constants and volumes of distribution in 13 obese vs. 13 normal weight subjects. Their results showed that gentamicin and tobramycin are distributed less to adipose tissue compared to other tissues, and mean Vd in obese subjects closely approximates the Vd seen in normal subjects, when using normalized body mass +40% of adipose mass as total weight in obese subjects [1]. Bauer at al studied aminoglycoside pharmacokinetics in two matched groups of 30 normal weight vs. 30 obese patients with documented Gram negative infection, and concluded that pharmacokinetics are altered in obesity, therefore morbidly obese patients require significantly higher gentamicin, tobramycin or amikacin doses compared to normal weight patients [5]. To compensate for increased Vd in obesity, aminoglycoside loading doses can be based on adjusted body weight (ABW), which is calculated using the formula: ABW = IBW + 0.4 × (TBW - IBW), where IBW is ideal body weight, and TBW is total body weight [5]. However, these studies were conducted at a time when once-daily antibiotic regimens (5 - 7 mg/kg for tobramycin or gentamicin), which are popular today, were not widely used. Analysis of pharmacokinetic data from 2073 patients (497 receiving tobramycin and 1,576 receiving gentamicin) using various formulas for Vd, showed that Vd was normalized for both tobramycin and gentamicin at all weight categories with use of lean body weight (LBW) estimated based as described by Janmahasatian et al [6, 7].

In 1995, Traynor et al investigated aminoglycoside pharmacokinetics in 1,708 patients who received gentamicin and tobramycin. Regression analysis revealed that the TBW to IBW ratio (TBW/IBW) predicted Vd. Furthermore, there were no large differences between dosing weight correcting factors (DWCFs) derived from IBW and body mass index (BMI)-based classification systems. This study concluded that, when calculating aminoglycoside doses, BMI values were not better than IBW, and proposed that DWCF for aminoglycoside doses should be IBW + 0.43 × EBW (where EBW is the excess body weight (EBW = TBW - IBW)) for patients with TBW/IBW ratio ≥ 1.25 [8].

In 1997, Wurtz et al published a review on antimicrobial pharmacokinetics and dosing in obesity, and proposed that, because 30% of adipose tissue is water, a DWCF of 0.3 should be used. Therefore the weight used to calculate doses of hydrophilic antibiotics is derived from the equation: IBW + 0.3 × (TBW - IBW), where IBW is derived from the Devine formula [9] and TBW is total body weight. However, other clinical data suggest that the DWCF is 0.4 for aminoglycosides [10]. Published data on aminoglycoside DWCFs are summarized in Table 1 [1-5, 8, 10-12].

**Table 1:** Summary, in Chronological Order, of Studies on Aminoglycoside Pharmacokinetics and Dose Adjustment in Obesity

Author, year	Antibiotic studied	Main findings
Schwartz et al, 1978 [1]	Gentamicin, tobramycin	Vd in obese subjects closely approximates Vd in normal subjects when using the formula: ABW = IBW + 0.4 × (TBW - IBW)
Blouin et al, 1979 [2]	Tobramycin	Tobramycin loading dose based on ABW = IBW + 0.58 × (TBW - IBW)
Korsager, 1980 [4]	Gentamicin	Gentamicin uptake in adipose tissue = 43.7% of uptake in total body mass of normal-weight patients
Bauer et al, 1980 [3]	Amikacin	Amikacin loading dose based on ABW = IBW + 0.38 × (TBW - IBW)
Bauer et al, 1983 [5]	Gentamicin, tobramycin, amikacin	Loading dose based on ABW = IBW + 0.4 × (TBW - IBW)
Leader et al, 1994 [11]	Gentamicin	Initial dose based on calculation of ClCr by Cockroft equation with IBW + 0.4 × (TBW - IBW)
Traynor et al, 1995 [8]	Tobramycin	The TBW/IBW ratio predicts Vd. For patients with TBW/IBW ratio ≥ 1.25, doses based on ABW = IBW + 0.43 × (TBW - IBW)
Wurtz et al, 1997 [10]	Aminoglycosides	DWCF = 0.4, therefore ABW = IBW + 0.4 × (TBW - IBW)
Pai et al, 2007 [12]	Aminoglycosides	Dosing of aminoglycosides should be based on ABW.

ABW: adjusted body weight; ClCr: creatinine clearance; DWCF: dosing weight correction factor; IBW: ideal body weight; TBW: total body weight; Vd: volume of distribution.

Our search revealed several reports describing physiologic changes in obesity [2, 10, 13, 14]. Because aminoglycoside dosing in critically ill obese patients has not been extensively studied, we could only find limited data, including a literature review by Erstad, which showed that circulatory changes in sepsis affect dosing and pharmacokinetics [15]. Data published by Cheymol support modification of loading and maintenance doses in critically ill obese patients due to physiologic alterations related to sepsis [16]. Although dose modification is described in studies by Leader, Corcoran and Blouin, appropriate aminoglycoside doses in critically ill obese patients are still debated [2, 11, 13, 17].

Regarding existing methods for measuring creatinine clearance (ClCr), published data provide accurate estimates for patients with normal weight, but not for obese ones. Compared to measured ClCr in obese patients, when the Cockroft-Gault equation is used with IBW, ClCr is underestimated, whereas ClCr is overestimated when TBW is used [18]. The Salazar-Corcoran equation, which shows strong correlation between ClCr and fat free body mass is as accurate as the Cockroft-Gault equation for the normal weight patients, but seems superior when applied to obese patients [19]. Therefore, when ClCr is not measured, estimation of ClCr using the Salazar-Corcoran equation should improve the ability to select appropriate doses for drugs cleared principally by renal filtration.

With regard to administration of aminoglycoside maintenance doses, most meta-analyses showed that once-daily regimens are equal or superior to multiple dose regimens, with regard to clinical efficacy, bacteriologic efficacy and nephrotoxicity [20-24].

## Discussion

Pathophysiologic changes in obesity affect most hydrophilic medications [10, 14, 25]. Because pharmacokinetic alterations in obesity affect absorption, Vd and clearance of many antibiotics, estimation of the correct antibiotic dose is of critical importance, especially in septic patients with multiple organ failure, where morbidity and mortality are high. However, despite obesity becoming a worldwide problem, antibiotic dosing in obese patients has not been studied adequately, and published data are very limited [26].

Morbid obesity alters aminoglycosides pharmacokinetics, as both Vd and clearance are increased in morbidly obese patients compared to patients with normal weight [16], but most published data in obesity refer to drug distribution. Pathophysiologic changes in obesity result in altered cardiac structure and function with increased cardiac output, altered tissue blood flow and increased gut perfusion. However, drug absorption does not seem to be altered, and several studies evaluating drug absorption did not find differences in obese vs. non-obese patients [10, 15]. At the present time, although Vd of many drugs, particularly lipophilic ones, is altered in obesity, there is no universally accepted factor to estimate Vd in obese individuals [27]. Although plasma proteins such as a1 acid-glycoprotein and lipoproteins are highly concentrated in obesity, binding of drugs to albumin does not seem to be altered. Vd of drugs equally soluble in water and oil (such as aminoglycosides) is slightly increased in obesity. Also, because distribution of a drug between adipose tissue and other tissues influences pharmacokinetics in obese patients, loading dose should be adjusted for IBW. Maintenance dosage also needs adjustment, depending on changes in drug clearance, as described in several studies on obese individuals [16]. Hepatic clearance of many drugs is also altered in obesity, but the metabolic capacity of the liver must be reduced > 90% before drug metabolism is significantly affected [25, 28].

In conclusion, Vd seems to be the pharmacokinetic variable most affected in obesity, and can be estimated using an ABW that includes a fraction of the excess body weight (TBW-IBW) [29].

ClCr, a useful measure for approximating glomerular filtration rate (GFR), is generally overestimated when it is calculated based on TBW, but is generally underestimated when based on IBW [2, 10]. The Cockcroft-Gault equation can be used to estimate GFR in lean patients, but its use in obesity is questionable due to disparity between muscle mass and body weight ratio [18]. The Salazar-Corcoran equation is an attempt to more accurately estimate ClCr in obesity, by taking into account serum creatinine, gender, TBW, age and height [19]. A comparison of the Cockroft-Gault vs. the Salazar-Corcoran equation showed that prediction of gentamicin elimination rate constant, clearance and elimination half time was best when ClCr was estimated using the Cockroft-Gault equation [11]. However, according to retrospective studies, the Salazar-Corcoran formula is more precise for obese patients [12, 30].

Aminoglycosides (gentamicin, amikacin, tobramycin, netilmicin, kanamycin and streptomycin) are cornerstones of therapy for serious Gram negative and aerobic bacilli infections [31], therefore appropriate aminoglycoside loading and maintenance dosing is important for good outcome. When initiating aminoglycoside therapy, a loading dose is needed to achieve therapeutic levels quickly. The loading dose is very important with aminoglycosides, because a high initial dose is needed to maximize their anti-bacterial effect and lower mortality [32, 33]. In addition, because aminoglycoside effect is concentration-dependent, administration of maintenance doses using extended interval regimens is preferred. Loading dose depends on Vd and target plasma concentration (Cp), both of which are affected by critical illness: Vd is altered in sepsis, due to alterations in microvascular permeability and abnormalities of extracellular body water. In one prospective study, Vd was estimated to be 0.43 ± 0.12 L/kg in the beginning of gentamicin therapy, but, as patient condition stabilized Vd was reduced to 0.29 ± 0.17 L/kg on the seventh day of treatment [34]. Because Vd and drug metabolism change with patient condition, optimization of aminoglycoside dosing is difficult in critical illness: hepatic and renal failure can affect antibiotic clearance, and critically ill patients have increased Vd and variable clearance compared to patients with less severe disease [35]. As Vd increases, aminoglycoside loading doses need to be increased in sepsis [15], and plasma level monitoring (also known as TDM) has been recommended to improve safety and effectiveness [34, 36, 37].

Aminoglycoside dosing in obese critically ill patients is even more challenging because drug distribution between fat and lean tissue influences pharmacokinetics, therefore aminoglycosides, being hydrophilic agents, need dosing adjustment. At the present time, there are limited data on the effects of fluid changes in critically ill obese ICU patients with sepsis [15], but the need for dose adjustment has been established for gentamicin, tobramycin and amikacin [2, 11, 17].

In healthy adults, aminoglycoside Vd is approximately 0.26 L/kg (range: 0.2 - 0.3). However, aminoglycoside dosing needs modification in obesity using a correcting factor of 0.4, because an estimated 40% of the dose is distributed into adipose tissue. [11, 15, 17]. Therefore, the equation for calculating ABW reads as follows: ABW = IBW + 0.4 × (TBW - IBW).

The above dosing modification is very important, because calculation of aminoglycoside dosing based on TBW may result in high serum concentrations, thereby increasing the risk for nephrotoxicity and ototoxicity [2, 16] in obesity. However, data regarding nephrotoxicity are conflicting, with studies on aminoglycoside kinetics showing no significant difference between obese patients and patients with normal weight with regard to ClCr for gentamicin [11].

Obesity is related to glomerular hyperfiltration, rather than to effects on tubular secretion [7]. The Cockcroft-Gault [18], the Salazar-Corcoran [19] and the modification of diet in renal disease (MDRD) [38] equation are the equations most commonly used for estimation of clearance. However, although these formulas are widely used, they are not reliable estimators of function, particularly in patients with high BUN/SCr ratio [39]. Furthermore, the predictive performance of newer formulas such as the MDRD and chronic kidney disease-epidemiology (CKD-EPI) [40] compared to the Cockcroft-Gault equation for assessment of kidney function and estimation of aminoglycoside clearance in obesity is unknown [41]. A study by Pai suggested that the CKD-EPI equation best predicts aminoglycoside clearance [7]. Overall, MDRD provides more reliable estimations of renal function compared to the Cockcroft-Gault formula, but both formulas lack precision. MDRD is not superior to Cockcroft-Gault for drug dosing, and MDRD estimated GFR (eGFR) needs adjustment to patient’s body surface area for drug dosing. Although Cockcroft-Gault has the advantage of simplicity and longer use [42], drug dosing guidelines are often based on ClCr estimated using the Cockcroft-Gault equation as surrogate for GFR.

Controversy exists with regard to the use of these formulas in obesity: the Cockcroft-Gault equation relies on TBW, and therefore overestimates GFR in obese patients. Similarly, the MDRD equation, which indexes GFR based on a normalized body surface area of mL/min/1.73 m^2^ also overestimates GFR in obese patients.

Aminoglycoside dosing is based on weight, according to either of two strategies: traditional, more frequent dosing, vs. extended-interval dosing, but dosing interval should be adjusted in patients with renal impairment. According to the American Society of Health Systems Pharmacists, in patients with normal renal function, traditional dosing consists of 1 - 2 mg/kg doses given multiple times per day, whereas the newer strategy utilizes 5 - 10 mg/kg every 24 h. Although aminoglycosides have been administered using multiple daily dosing regimens for decades, newer clinical and laboratory studies suggest that once-daily dosing confers advantages with regard to efficacy [43, 44]. With regard to extended interval dosing in obesity, a retrospective analysis by Ross suggests that use of DWCF of 40% is accurate [45].

In ICU patients, however, other factors such as sepsis and renal dysfunction can significantly alter aminoglycoside pharmacokinetics, and individual TDM is warranted [46]. Details regarding the effects of fluid shifts on aminoglycoside pharmacokinetics have not been well studied in critically ill obese patients. Finally, the advantages of large once-daily aminoglycoside doses have been questioned, because of apparent pyrogen-mediated toxicity [47]. As relevant published data are very limited [47], there is great need for well-conducted clinical trials on obese critically ill patients [15].

Understanding of the pathophysiologic changes in obesity and knowledge of the literature on aminoglycosides are valuable for rational determination of dosages in obese patients. As aminoglycosides are first-line drugs in sepsis, there is clearly a need for more studies on the appropriate use and dosing of aminoglycosides in critically ill obese patients. It is important to realize that studies on pharmacokinetics in obesity have limitations, such as patient population heterogeneity, small patient numbers, the fact that many data are referring to single doses rather than ongoing use, and limited knowledge on the effects of obesity on pharmacokinetics. Failure to appropriately modify doses in obesity can result in therapeutic failure or increased toxicity, therefore TDM, when feasible, has been recommended in order to optimize the safety and effectiveness of aminoglycoside antibiotic therapy [37, 48].

## Conclusion

Modification of drug dosage in obese patients is very important, particularly when using medications with narrow therapeutic index. Alterations of aminoglycoside pharmacokinetic parameters in obesity may necessitate deviation from dosages commonly recommended for non-obese individuals, but knowledge on the influence of obesity on pharmacokinetics is limited. This review shows that currently available literature supports the need for aminoglycoside dosage modification in critically ill obese patients. Physicians need to take every possible step to ensure that aminoglycoside doses are adequate and safe. Because pathophysiologic changes in obesity can result in sub- or supra-therapeutic concentrations, aminoglycoside doses should be re-evaluated daily in critically ill obese patients, and TDM can help optimize therapy. Clinicians prescribing antibiotics for critically ill obese patients should be familiar with drug and patient factors influencing the effectiveness of antibiotic therapy.
